# Editorial: Trematode Infection in Ruminants

**DOI:** 10.3389/fvets.2021.719577

**Published:** 2021-07-14

**Authors:** Theo de Waal, Khalid Mehmood

**Affiliations:** ^1^School of Veterinary Medicine, University College Dublin, Dublin, Ireland; ^2^Faculty of Veterinary and Animal Sciences, The Islamia University of Bahawalpur, Bahawalpur, Pakistan

**Keywords:** trematode infection, *Fasciola*, *Paramphistomum*, *Schistosoma*, one health-approach

Trematodes are a diverse group of parasites affecting both humans and animals health worldwide. One school of thought is that trematodes evolved from free-living flatworms (progenitors of present-day rhabdocoel turbellarians), became intimately associated with mollusks and, ultimately, developed into parasitic forms. Evolutionary divergence within this endoparasitic population gave rise to two subclasses, the Digenea and Aspidobothrea (1). The Digenea is one of the largest groups of platyhelminths and parasitises a wide range of invertebrate and vertebrate hosts which also includes humans. Within the vertebrate, final host, these worms are found in numerous organs, including the intestine, lungs, liver, and vascular system. Infections with these parasites are responsible for substantial production losses in the livestock industry and decrease in the quality-of-life in humans (2–6).

Fasciolosis causes substantial economic losses to pastoral agricultural communities and commercial animal farmers, estimated at US$ 2 billion per year, through the death of infected cattle, liver condemnation, and productivity losses associated with reduced feed conversion quality. Fasciolosis is widespread in tropical areas, affecting up to 90% of cattle, and is considered the most significant helminth parasite (7). *Fasciola hepatica* infections are reported to affect weight gain, milk production, milk solids content and fertility of cattle (8, 9). More recent studies provided sound evidence that milk production in dairy cattle or carcass weight of slaughtered beef cattle in *F. hepatica*-infected herds was decreased on average by 3–5% or 0.5–0.7%, respectively (10, 11).

Furthermore, fasciolosis is recognized as emerging disease in humans. The World Health Organization has anticipated that 180 million people are at risk of infection and 2.4 million people are infected with fasciolosis in more than 60 countries (https://www.who.int/foodborne_trematode_infections/fascioliasis/en/). Fasciolosis in people is responsible for a decrease in the quality of life with the global burden of food-borne fasciolosis estimated 90,041 Disability Adjusted Life Years (12). The northern Bolivian Altiplano is one of the areas where the highest prevalence of *F. hepatica* in humans have been reported (13). Even at this high altitude (>3,200 m above sea level) the parasite is capable of completing its life cycle with infection levels of 20.6–63.1% in cattle and sheep, respectively (Mas-Coma et al.).

With the increasing reports of anthelmintic resistance in *F. hepatica* (14) there are many research studies underway to find alternative control option including vaccination (15) and alternative drug options (14). One such option is protein kinase inhibitors which has been widely studied in *Schistosoma* spp. In the paper of Morawietz et al. the authors investigated two kinase inhibitors used in human cancer research: the Abelson tyrosine kinase (ABL-TK) inhibitor imatinib and the polo-like 1 (PLK1) inhibitor BI2536 as therapeutic targets in *F. hepatica* and have shown that both inhibitors had lethal effects on immature flukes but only imatinib was lethal in adult flukes. The results of phylogenetic analysis support the logical evolution of fasciolosis transmitted by planorbid snails, beginning in Africa and spreading to Asia and the rest of the Holarctic Region (16).

Although paramphistomes (rumen flukes) are generally considered of less importance in animal health there has been an increase incidence of infections in both cattle and sheep, in recent years, especially in Europe (17), sometimes leading to dramatic outbreaks (18, 19). More than 70 species of paramphistomes infecting animals, has been described but distinguishing between species morphologically is difficult and requires expertise. Accurate species identification is important if we aim to understand the epidemiology and host-parasite interaction of this diverse group of parasites better. Mitchell et al. described a universal approach to rumen fluke identification across species and hosts using molecular techniques. Schols et al. argue for the use of cytochrome c oxidase subunit I (COI)-based barcoding as an important key part of the interagive taxonomy for trematodes and they successfully identified trematodes ion both the snail intermediate host as well as in the definitive animal host.

Overall, this Research Topic provide a brief review of the current research relating to trematode infection ultimately improving our understanding and providing new strategies to control trematode diseases.

## Author Contributions

TW wrote the first draft of the manuscript. KM revised and added additional text to the final manuscript. All authors contributed to the article and approved the submitted version.

## Conflict of Interest

The authors declare that the research was conducted in the absence of any commercial or financial relationships that could be construed as a potential conflict of interest.
